# Characterization of a newly isolated strain *Pseudomonas* sp. C27 for sulfide oxidation: Reaction kinetics and stoichiometry

**DOI:** 10.1038/srep21032

**Published:** 2016-02-11

**Authors:** Xi-Jun Xu, Chuan Chen, Hong-liang Guo, Ai-jie Wang, Nan-qi Ren, Duu-Jong Lee

**Affiliations:** 1 State Key Laboratory of Urban Water Resource and Environment, Harbin Institute of Technology, P. O. Box 2614, 202 Haihe Road, Harbin 150090, China; 2 Department of Chemical Engineering, National Taiwan University, Taipei 10617, Taiwan

## Abstract

Sulfide biooxidation by the novel sulfide-oxidizing bacteria *Pseudomonas* sp. C27, which could perform autotrophic and heterotrophic denitrification in mixotrophic medium, was studied in batch and continuous systems. *Pseudomonas* sp. C27 was able to oxidize sulfide at concentrations as high as 17.66 mM. Sulfide biooxidation occurred in two distinct stages, one resulting in the formation of sulfur with nitrate reduction to nitrite, followed by thiosulfate formation with nitrite reduction to N_2_. The composition of end-products was greatly impacted by the ratio of sulfide to nitrate initial concentrations. At a ratio of 0.23, thiosulfate represented 100% of the reaction products, while only 30% with a ratio of 1.17. In the continuous bioreactor, complete removal of sulfide was observed at sulfide concentration as high as 9.38 mM. Overall sulfide removal efficiency decreased continuously upon further increases in influent sulfide concentrations. Based on the experimental data kinetic parameter values were determined. The value of maximum specific growth rate, half saturation constant, decay coefficient, maintenance coefficient and yield were to be 0.11 h^−1^, 0.68 mM sulfide, 0.11 h^−1^, 0.21 mg sulfide/mg biomass h and 0.43 mg biomass/mg sulfide, respectively, which were close to or comparable with those reported in literature by other researches.

Sulfide production is often observed in sulfidogenic treatment of acid mine drainage, gasification of coal for electricity, and petrochemical industry^1^,^2^,^3^. The toxic, corrosive and odorous nature has motivated a growing interest to use various strategies to control sulfide associated problems. Compared with physicochemical processes such as Claus, Alkanolamine, Lo-Cat and Holmes-Streford^4^, biological treatments relying on sulfide-oxidizing bacteria are cost-effective and environmental friendly, which are operating at ambient pressure and temperature without the need for expensive biocatalysts and feasible for the removal of low levels of sulfide^5^,^6^. In the presence of a suitable electron acceptor, sulfide-oxidizing bacteria could oxidize sulfide to sulfur or sulfate thereby decreasing the level of sulfide in the wastewaters. The use of nitrate has been proven to be very effective and originally attributed to biological oxidation of sulfide by autotrophic denitrifiers (nitrate-reducing, sulfide-oxidizing bacteria, NR-SOB) such as *Thiomicrospira denitrificans*, and some strains of *Thiomicrospira* sp., *Thiobacillus* sp., and *Acrobacter* sp.^7^,^8^,^9^,^10^,^11^. Banking on the fact of the occurrence of autotrophic denitrification when organic matter is present^12^,^13^,^14^,^15^ and the general existence of organic matter in the wastewaters, a biological process based on the manipulation of heterotrophic nitrate-reducing bacteria (h-NRB) and NR-SOB has been developed. In the synergetic system, sulfide is firstly oxidized to elemental sulfur with nitrate reduction to nitrite by NR-SOB and subsequently the formed nitrite is reduced to nitrogen gas (N_2_) in the expense of organic carbon oxidation by h-NRB^15^.

Despite the substantial amount of research on biooxidation of sulfide under denitrifying conditions^5^,^12^,^13^,^16^,^17^,^18^,^19^,^20^,^21^, the sensitivity of bacteria to change in operating factors such as high levels of sulfide, sulfide to nitrate ratio, and organic carbon to nitrate ratio has restricted the widespread application of this technology. Recent work on the microbiology of sulfide oxidation under denitrifying conditions has led to the isolation and identification of a novel sulfide-oxidizing bacterium designated as *Pseudomonas* sp. C27 from a denitrifying sulfide removal reactor^22^, which can grow on heterotrophic and mixotrophic medium and perform both autotrophic and heterotrophic denitrification in mixotrophic medium. The capability of *Pseudomonas* sp. C27 to use nitrate as a terminal acceptor for sulfide oxidation and acetate oxidation makes it a suitable candidate for simultaneous removal of sulfide, nitrate and acetate under anaerobic conditions. Preliminary studies have concentrated mainly on the physiological and microbiological aspects^22^.

Considering the limited amount of work on biooxidation of sulfide under denitrifying conditions by *Pseudomonas* sp. C27, and the important role which this bacterium could play in treatment of sulfide-laden wastewaters, further research is essentially required to explore the potential of this novel biocatalyst. The present work studies the effects of initial concentrations of sulfide, molar ratios of sulfide to nitrate and carbon to nitrate on the activity of *Pseudomonas* sp. C27 and composition of sulfide biooxidation end products, and the data collected in the batch tests provides insight regarding the kinetics of sulfide biooxidation by *Pseudomonas* sp. C27. Additionally, sulfide biooxidation by *Pseudomonas* sp. C27 in continuous system is also evaluated herein.

## Results and Discussion

### Batch experiments

In the control tests maintained under anaerobic conditions without inoculums, initial decreases in concentrations of sulfide were observed. With 5.6 mM sulfide, the initial decrease amounted to 1.8% of the total sulfide present. No obvious changes in sulfate and nitrite concentrations were observed throughout the abiotic experiments. The initial decrease in sulfide concentration could be attributed to either transfer of sulfide from the liquid phase to the head space gas or abiotic oxidation of sulfide, which was in accordance with results obtained by An *et al.*^5^ with *Thiomicrospira* sp. CVO as inoculum.

**Figure 1:**
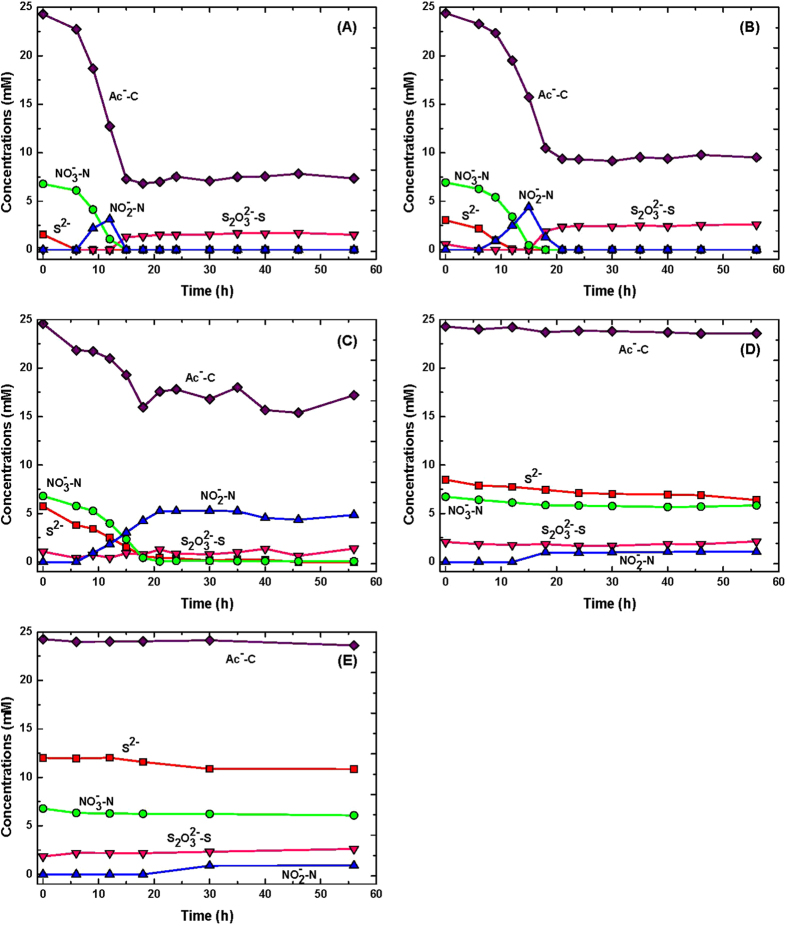
Profiles of sulfide, thiosulfate, nitrate, nitrite and acetate concentrations in the batch cultures of *Pseudomonas* sp. C27 with 7.5 mM nitrate, 11.25 mM acetate and various initial sulfide concentrations: (**A**) 1.56, (**B**) 3.13, (**C**) 5.63, (**D**) 8.50, and (**E**) 12.02 mM.

Figure 1 presents selected results obtained in the batch experiments aiming to verify the effects of sulfide initial concentration. With 1.56 mM of sulfide, the sulfide concentration reached to a negligible value in less than 6 h (removal rate: 0.26 mM/h). In all cases, biooxidation occurred in two distinct stages. First, sulfide concentration decreased continuously but thiosulfate concentration remained constant. Once concentration of sulfide reached to a low level, a continuous increase in concentration of thiosulfate was observed. During this phase the turbidity became clear, an indication of oxidation of sulfur to thiosulfate. Sulfide oxidation was accompanied by denitrification, which also occurred in two distinct phases, coinciding with removal of sulfide (oxidation of sulfide to sulfur with nitrate reduction to nitrite) and production of thiosulfate (oxidation of sulfur to thiosulfate coupling nitrite reduction to N_2_ gas). Reduction of nitrate leading to production of nitrite was observed during the whole experiments. With 1.56 and 3.13 mM sulfide, the levels of produced nitrite were 3.13 and 4.39 mM, respectively and nitrite was then utilized as bio-oxidant proceeded for sulfur oxidation to thiosulfate. With 5.63 mM sulfide, concentration of nitrite decreased slowly from an initial value of 5.25 to 4.36 mM at the end of experiment. A small amount of sulfate (~0.1 mM) was detected in the samples taken immediately after the inoculation. It seems that sulfate was not an end product for sulfide oxidation as the level of present sulfate remained unchanged (data not shown). A decrease in concentration of acetate was observed at the end of experimental runs. This decrease could be due to slightly heterotrophic denitrification and microbial growth. As shown by Chen *et al.*^22^, *Pseudomonas* sp. C27 could not grow when carbonate was used as the sole carbon source. With 12.02 mM sulfide, apart from an initial decrease of 1 mM, potentially due to spontaneous chemical oxidation, sulfide oxidation remained constant even after a prolonged period of monitoring. Nitrate and acetate concentration also remained constant and no nitrite was detected, indicating that the bacteria were not active. Sulfide removal rates in the culture initially containing 1.56, 3.13, 5.63 and 8.50 mM sulfide were 0.26, 0.41, 0.65 and 0.10 mM h^−1^, respectively. The extended lag phase and lower oxidation rate obtained with 8.50 mM sulfide, and inability of bacteria to oxidize 12.02 mM sulfide indicate sulfide inhibition effect. The nitrate removal rates in the culture containing 1.56, 3.13, 5.63 and 8.50 mM sulfide were 1.01, 0.98, 0.64 and 0.10 mM h^−1^, respectively. With 1.56 and 3.13 mM sulfide, nitrite removal rates of 0.59 and 0.84 mM h^−1^ observed during the production of thiosulfate, respectively. With 5.63 mM sulfide, nitrate was used completely via both sulfide-driven denitrification and heterotrophic denitrification, and the produced nitrite at a concentration up to 5.3 mM accumulated in the system until the end of experiment indicating an inhibition effect of nitrite on sulfur oxidation to thiosulfate coupled with nitrite reduction to N_2_ gas.

**Figure 2:**
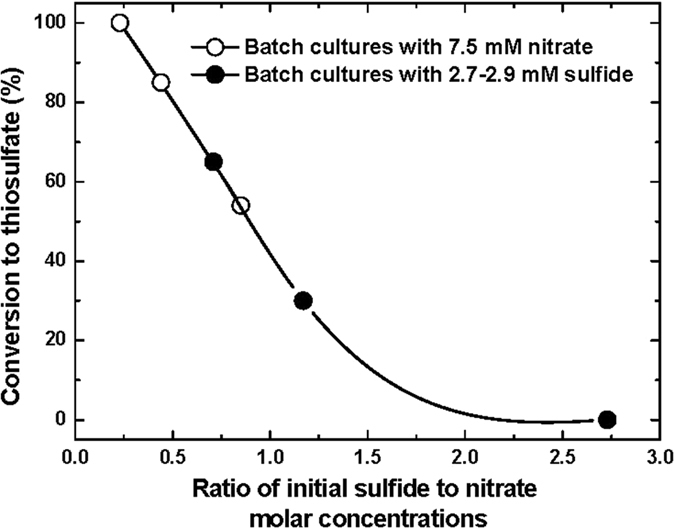
Effect of initial sulfide to nitrate molar concentrations on the conversion of sulfide to thiosulfate in the batch cultures of *Pseudomonas* sp. C27; data derived from batch cultures with 7.5 mM nitrate and 2.7–2.9 mM sulfide.

In order to establish a criterion for controlling the composition of end-products, the extent of thiosulfate formation was related to the initial ratio of sulfide to nitrate concentrations. The information compiled in Fig. 2 shows that at low values of sulfide to nitrate ratio, thiosulfate was the main product and as this ratio was increased the conversion of sulfide to thiosulfate was decreased. For instance, at a ratio of 0.23, thiosulfate constitutes almost 100% of the end products for sulfide oxidation, while with a sulfide to nitrate ratio of 1.17 the conversion of sulfide to thiosulfate was only around 30%. When sulfide to nitrate ratio was up to 2.7 no thiosulfate was detected during the whole experiment. This was consistent with information in the literature regarding the effects of sulfide to nitrate ratio on the oxidation state of sulfur compounds, although the end-products of sulfide biooxidation by *Pseudomonas* sp. C27 was generally different from other sulfide-oxidizing species (e.g. *Thiobacilli* species, *Thiomicrospira* species). Using a pure culture of *Thiobacillus denitrificans* strain D-4, Wang *et al.*^21^ found that both the initial sulfide concentration and sulfide to nitrate ratio had a strong impact on the formation of intermediate- and final oxidation products. The optimal influent sulfide concentration and sulfide to nitrate molar ratio were suggested be less than 9 mM sulfide and in the range of 1.6–2.5, respectively. Cardoso *et al.*^16^ studied the effect of nitrate concentrations on chemolithotrophic denitrification and demonstrated that partial oxidation from sulfide to elemental sulfur was driven by a limitation of electron acceptor. Using *Thiomicrospira* sp. CVO, Gadekar *et al.*^19^ also shown that at low values of sulfide to nitrate ratio (0.28), sulfate (93% of the reaction product) was the main product. Similarly, using a mixed-culture from the produced water of Coleville oil field in Canada dominated by *Thiomicrospira* sp. CVO, An *et al.*^5^ revealed that when at the lowest ratio of 0.2 sulfate was the sole end product, while with the highest ratio of 3.1 only 4.4% of sulfide was converted to sulfate. Likewise the same criterion for controlling the composition of end-products with oxygen as electron acceptor was observed by Buisman *et al.*^23^, Janssen *et al.*^24^, Alcantara *et al.*^25^, and Xu *et al.*^26^.

**Figure 3:**
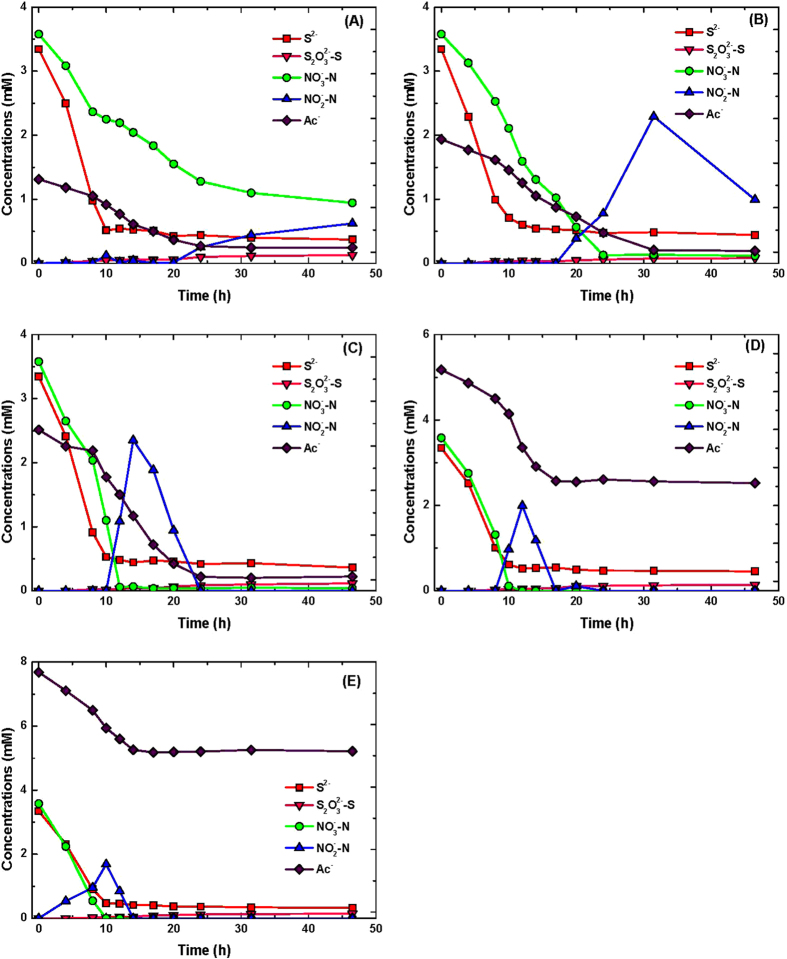
Profiles of sulfide, thiosulfate, nitrate, nitrite and acetate concentrations in the batch cultures of *Pseudomonas* sp. C27 with 3.34 mM sulfide, 3.58 mM nitrate and various initial acetate concentrations: (**A**) 1.31, (**B**) 1.94, (**C**) 2.51, (**D**) 5.17, and (**E**) 7.68 mM.

Selected results representing the effect of carbon to nitrate ratio were shown in Fig. 3. With ratios in the range 0.75–5.0 sulfide removal rates remained nearly constant (0.38 mM h^−1^) indicating that sulfide biooxidation by *Pseudomonas* sp. C27 was not greatly affected by carbon to nitrate ratio. The rapid conversion of sulfide might be explained by the low electron equivalent requirement for the oxidation of sulfide to elemental sulfur coupled to the reduction of nitrate to nitrite or N_2_. In all cases nitrate reduction and concomitant production of nitrite occurred during the removal of sulfide. The reduction rate of nitrate increased from 0.18 to 0.60 mM h^−1^ as carbon to nitrate ratio increased from 0.75 to 3. For ratios of 0.75 and 1.26, higher levels of nitrite were produced and accumulated in the system till the end of the experiments as expected. While with ratios in the range of 1.63 to 5 nitrite was eventually reduced but only after complete utilization of nitrate, indicating that nitrate was the preferred electron acceptor for the bacteria. A continuous but very slow increase of thiosulfate was observed in all cases, suggesting a slow rate of sulfur oxidation to thiosulfate with nitrite reduction to N_2_ and with excess organic carbon present, *Pseudomonas* sp. C27 prefers the use of acetate for nitrite reduction than the use of sulfur.

Biooxidation of sulfide to elemental sulfur in the presence of nitrate could occur through two different reactions outlined below:


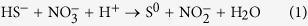



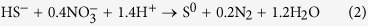


The stoichiometry of Reaction 1 indicated that oxidation of each mole of sulfide results in formation of one more nitrite. Using the experimental data (3.13 mM sulfide, 7.5 mM nitrate and 11.25 mM acetate), sulfide biooxidation and nitrate reduction rates were determined to be 0.146 and 0.108 mM h^−1^, respectively. This together with a constant thiosulfate concentration during the biooxidation of sulfide, and the presence of suspended particles in the culture implied that during this phase sulfide was mainly oxidized to elemental sulfur with concomitant reduction to nitrite via Reaction 1. Formation of thiosulfate was observed as soon as the sulfide and nitrate concentration reached a negligible level. The turbidity of the culture decreased, indicating the oxidation sulfur to thiosulfate. Based on the experimental data collected during thiosulfate formation, the rates of thiosulfate production, nitrite production followed by nitrite reduction were 0.63, 0, −1.04 mM h^−1^, and as a result biooxidation of sulfur to thiosulfate must have occurred with nitrite reduction to N_2_ (Reaction 3).


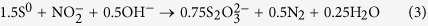


### Continuous experiments

**Figure 4:**
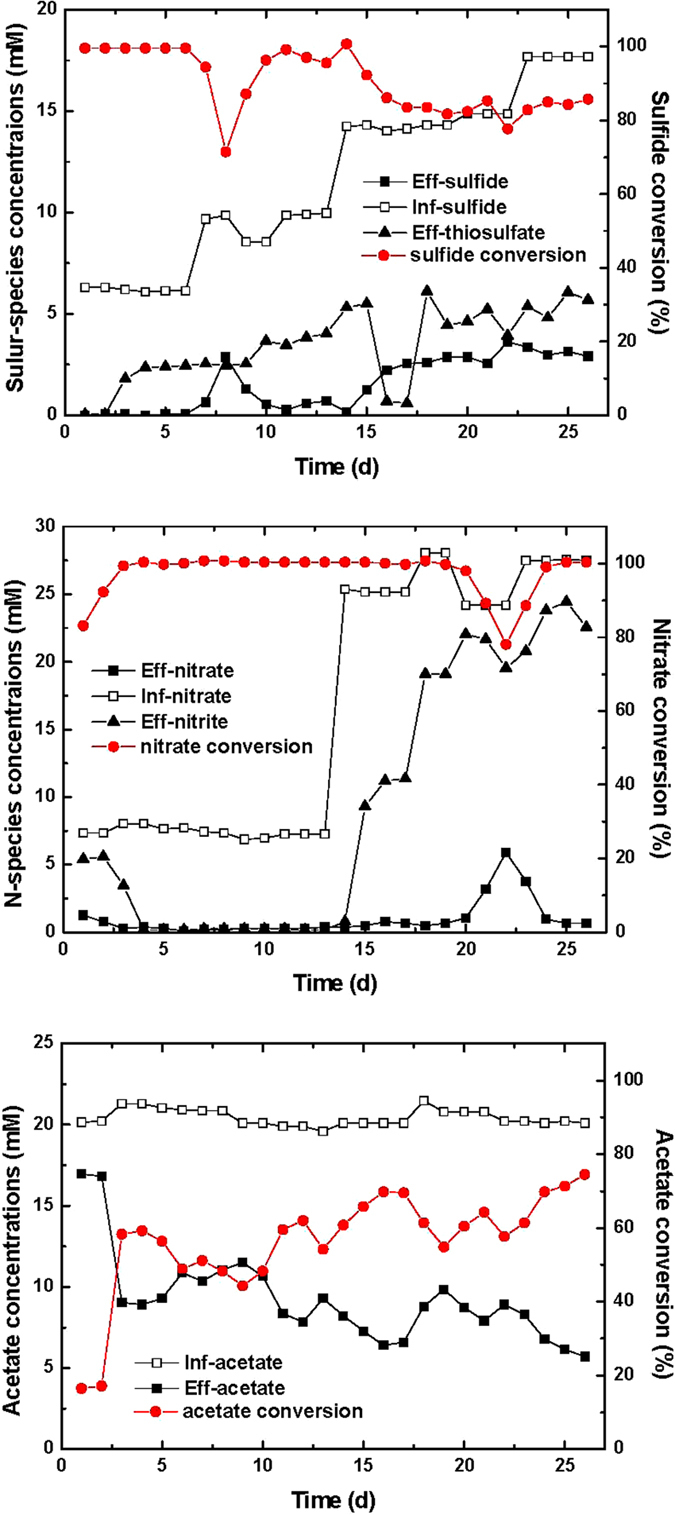
Profiles of sulfide, thiosulfate, nitrate, nitrite and acetate concentrations in the continuous experiments with *Pseudomonas* sp. C27.

The steady-state profiles of residual sulfide, thiosulfate, nitrate, nitrite and acetate concentrations observed were shown in Fig. 4. With 6.25 and 9.38 mM sulfide, residual sulfide and nitrate in the effluent reached a negligible level, with the formed thiosulfate concentrations being 2.45 and 4.03 mM, respectively. Further increase in initial sulfide concentration led to a slightly decrease in sulfide removal efficiency (~85%) indicating a sulfide inhibition effect. Nevertheless, the tolerance of sulfide by *Pseudomonas* sp. C27 was improved in the continuous reactor compared to that in batch experiments. With sulfide concentration up to 17.66 mM, more than 80% of sulfide biooxidation was achieved. The concentration of sulfate remained relatively low over the entire range of initial sulfide concentrations, indicating that sulfate might not be the end-product of oxidation of sulfide. This was consistent with the data compiled from the batch experiments (Fig. 1). Oxidation of sulfide led to formation of nitrite, with high nitrite concentrations observed at high initial sulfide concentrations (14.06 and 17.66 mM). And this might be due to the sulfide inhibition effect on heterotrophic denitrification.

### Kinetics of sulfide biooxidation by *Pseudomonas* sp. C27

Our approach in development of a kinetic model for biooxidation of sulfide (nitrate and acetate in excess) by *Pseudomonas* sp. C27 was based on the fundamental relationships between the kinetics of bacteria growth and anaerobic biooxidation of sulfide. For a growth-associated biological reaction, the rate of substrate utilization (sulfide biooxidation in this case) can be shown as follows:


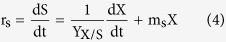



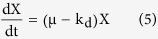


where r_s_ is the rate of substrate utilization (biooxidation rate of sulfide) (mM sulfide h^−1^), Y_X/S_ is the yield coefficient (mg biomass mmol^−1^ sulfide), 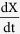 is rate of biomass (*Pseudomonas* sp. C27) formation (mg biomass h^−1^), m_s_ is the maintenance coefficient (h^−1^), μ is the specific growth rate (h^−1^), k_d_ is decay coefficient (h^−1^), and X is the biomass concentration (mg biomass L^−1^). Using the protein concentration and residual concentration of sulfide, determined at batch experiment with 5.63 mM initial sulfide, it was possible to describe the yield coefficient (Y_X/S_) of the bacterium. The values of yield and maintenance coefficient were determined to be 0.43 mg biomass/mg sulfide and 0.21 mg sulfide/mg biomass h, respectively.

The specific growth rate was treated with an unstructured, non-segregated model, Monod expression (eq. 6):


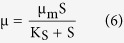


where μ_m_: the maximum specific growth rate (h^−1^), S: the limiting substrate concentration (sulfide in this case) (mM), and K_S_: the half saturation constant (mM).

**Figure 5:**
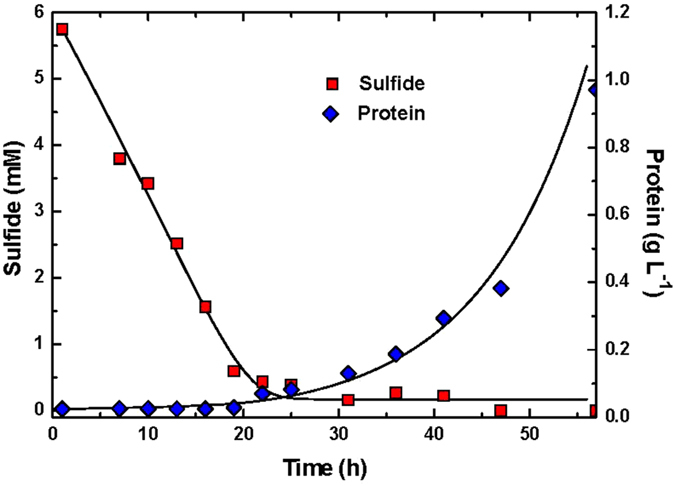
Model fitting results of sulfide and protein concentration profiles by *Pseudomonas* sp. C27 fed with medium containing 5.75 mM sulfide and 6.8 mM nitrate, 24.6 mM acetate.

**Figure 6:**
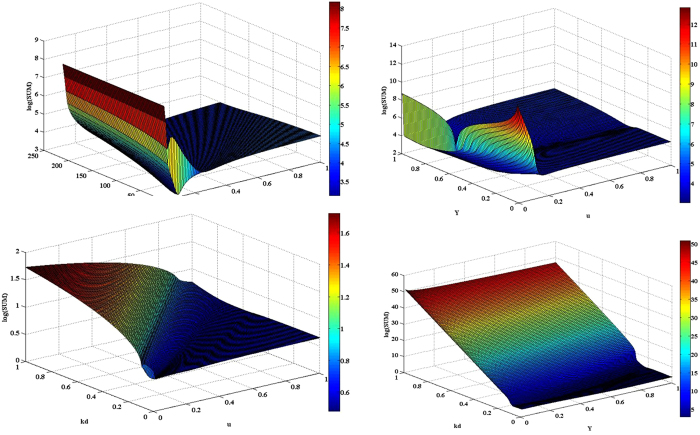
Surface plots of the objective function used for sulfide biooxidation by *Pseudomonas* sp. C27 parameter estimation (SUM) as a function of different parameter combinations: μm vs Ks (**A**); μm vs kd (**B**); μm vs Y (**C**); Y vs kd (**D**). The plots were drawn using the optimal parameters (Table 1) as midpoint of intervals with one order of magnitude change (except Y, which was always lower than 1) on both sides of intervals.

Using a non-linear regression program the values of kinetic coefficients were determined shown in Table 1. The modeling results profiles were included as solid lines in Fig. 5, showing a good agreement between simulated and experimental results. As a validation, the resulting parameter values were used to generate two additional sets of curves for comparison with two other data sets (1.56, 3.13 mM initial sulfide concentrations in batch experiments), again showing good correspondence to experimental data (results not shown). Parameter identifiability was investigated to tell which parameter combinations could be estimated under given measurement accuracy and quantity. In the identifiability analysis, surface plot of the sum of residual squares (eq. 7) for the degree of correlation between parameters were evaluated. The surface plots in Fig. 6 were calculated around the optimum for different combinations of parameters. The plots were drawn by the methods described in Ni *et al.*^27^. The surface of the objective function for half saturation constant (K_S_) and maximum specific growth rate (μ_m_) showed a well-defined valley, where the optimum values of μ_m_ and K_S_ resided. This indicated a good identifiability of these parameters. Generally, there was a greater sensitivity of the kinetics to μ_m_ compared to K_S_. There was a greater change in SUM on the μ_m_ compared with that on the K_S_.


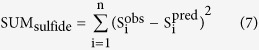


The literature regarding the kinetics of growth and sulfide biooxidation by *Pseudomonas* sp. C27 was very limited and prior to the present work no data for kinetic parameters have been reported for this bacterium. The maximum specific growth rate of *Pseudomonas* sp. C27, 0.11 h^−1^, was slightly lower than that reported for *Thiomicrospira* sp. CVO (0.36 h^−1^), however in accordance with those reported for *Thiobacilli* species (0.1–0.2 h^−1^). The half saturation constant of 0.68 mM determined for *Pseudomonas* sp. C27 was much lower than the reported values of 2 mM for *Thiomicrospira* sp. CVO, while slightly higher than that of 0.28 mM for *Thiobacilli*^19^,^25^. Assuming that the content of protein in biomass is roughly 50%^4^, the yield coefficient of 0.43 mg biomass/mg sulfide for *Pseudomonas* sp. C27 was relatively close to a value of 0.56 g biomass/mg sulfide for *Thiomicrospira* sp. CVO reported by Gadekar *et al.*^19^, and 0.375 mg biomass/mg sulfide determined by Sublette and Sylvester^28^ for *Thiobacillus denitrificans*.

The balanced growth of both autotrophic and heterotrophic denitrifiers is of crucial importance for long term stability of denitrifying sulfide removal process. However, it is not easy to achieve due to the different growth rate between heterotrophs and autotrophs. *Pseudomonas* sp. C27, which could conduct both autotrophic and heterotrophic denitrification, provides a potential to settle the “uneasy” and with a single culture (e.g. *Pseudomonas* sp. C27) no competition of its own growth arises in the bioreactor^22^. Considering the ability of *Pseudomonas* sp. C27 in oxidizing sulfide at concentrations as high as 18 mM (based on the results in continuous bioreactor), and the feasibility to a wide range of sulfide to nitrate and carbon to nitrogen molar ratio makes *Pseudomonas* sp. C27 a favorable biocatalyst for biooxidation and removal of sulfide in a variety of applications.

## Conclusions

The results of the present study revealed that *Pseudomonas* sp. C27 is capable of oxidizing sulfide at concentrations as high as 18 mM under anaerobic conditions in continuous bioreactors. Biooxidation of sulfide by *Pseudomonas* sp. C27 results in formation of sulfur or thiosulfate, and the ratio of sulfide to nitrate initial concentrations have a strong impact on the oxidation state of the end products. However, the ratio of carbon to nitrate initial concentrations seems to have little effect on sulfide biooxidation by *Pseudomonas* sp. C27. The kinetic parameters for the growth of *Pseudomonas* sp. C27 were determined in present work, which were in the same magnitude with those reported for *Thiomicrospira* sp. CVO and *Thiobacillus denitrificans*. Capability of conducting both autotrophic and heterotrophic denitrification, tolerance of high sulfide concentrations, adaptable to wild range of sulfide/nitrate/acetate molar ratio are characteristics of *Pseudomonas* sp. C27 which makes it a favorable biocatalyst for biooxidation and removal of sulfide. Field tests are required in future to test the possibility of the enhancement of sulfide removal through utilization of *Pseudomonas* sp. C27 immobilized cells.

## Materials and Methods

### Microbial culture and mediumh

A pure culture of *Pseudomonas* sp. C27 (GeneBank accession number GQ241351) was kindly provided by Dr. D. J. Lee, University of National Taiwan, Taipei, and was used in this study. The liquid medium used for the growth and maintenance of *Pseudomonas* sp. C27 was detailed as described by Chen *et al.*^22^. The medium made with reverse osmosis water contained: 19.87 mM NaCH_3_COO, 0.4 mM MgSO_4_·7H_2_O, 5.95 mM NaHCO_3_, 18.69 mM NH_4_Cl, 17.33 g/L KNO_3_, 13.2 mM KH_2_PO_4_, 6.9 mM K_2_HPO_4_ and 1 ml of trace element solution. The trace element solution contained: 134 mM EDTA, 275 mM NaOH, 50 mM CaCl_2_·2H_2_O, 18 mM FeCl_2_·4 H_2_O, 15.4 mM MnCl_2_·2H_2_O, 7.8 mM ZnCl_2_, 1.8 mM CoCl_2_·6H_2_O, 0.4 mM (NH_4_)_6_Mo_7_O_24_·4H_2_O, and 0.8 mM CuCl_2_·2 H_2_O. All medium components, except sodium sulfide, were combined and the pH was adjusted to 7.5, using 1 M HCl. Serum bottles (100 mL total volume) containing 50 mL medium were purged with nitrogen for 5 min, sealed and autoclaved for 30 min at 121 ^o^C. Filter sterilized stock solution of Na_2_S·9H_2_O (1 M) was added to the medium to a final concentration of around 6.25 mM and pH was readjusted to 7.5. A stock culture of *Pseudomonas* sp. C27 was used as inoculums (10% v/v). The cultures were maintained at 30 ^o^C and subcultured on a weekly interval.

### Batch Experiments performed

The effect of initial sulfide concentration on the activity of *Pseudomonas* sp. C27 was studied in 250 mL flasks each containing 200 mL of the liquid medium described above with 7.5 mM nitrate and 1.56, 3.13, 5.63, 8.50 or 12.02 mM sulfide. As *Pseudomonas* sp. C27 could not conduct autotrophic sulfide biooxidation without acetate present^22^, 11.25 mM acetate was also added in the medium to maintain the activity of *Pseudomonas* sp. C27. A 2-day-old *Pseudomonas* sp. C27 culture was used as an inoculum (10% v/v). All experiments were carried out at 30 ^o^C and sampled regularly anaerobically, which involved syringe injection of N_2_ to maintain pressure, prior to removing a liquid sample. Sulfide concentration was determined immediately after sampling. The remaining portion of the sample was centrifuged for 10 min at 8000 rpm and the supernatant was preserved in a freezer (−4 ^o^C) for further analysis. Concentrations of sulfate, thiosulfate, nitrate, nitrite and acetate were monitored in these samples during the course of the experiments. Protein concentration (as an indication of biomass concentration) was monitored in experiments with 5.63 mM initial sulfide.

The effect of sulfide to nitrate molar concentrations ratio (5/2, 5/6, 5/8) on sulfide oxidation, denitrification and composition of the end products was determined by conducting additional batch experiments in 250 mL flasks containing 200 mL sterilized medium with 1, 2.5 or 4.2 mM nitrate. The initial sulfide concentration in all bottles was adjusted to 2.7–2.9 mM and pH was set at 7.5. Bottles were inoculated with 20 mL (10% v/v) of a two-day-old *Pseudomonas* sp. C27 culture. The exact sulfide to nitrate ratios after adjustment of pH and inoculation were 2.73, 1.17 and 0.71. Additionally, 2.4 mM acetate was present in the medium. To assess the effect of acetate to nitrate molar concentrations ratio on sulfide oxidation under denitrifying conditions by *Pseudomonas* sp. C27, a number of experiments were carried out using mixotrophic medium containing 3.34 mM sulfide, 3.58 mM nitrate and 1.31, 1.94, 2.51, 5.17 or 7.68 mM acetate. All other conditions and monitoring approaches were similar to those described earlier. All batch tests were carried out in duplicate and average values were plotted in the figures. Control runs were conducted under similar conditions without inoculums.

### Continuous experiments: Effect of sulfide concentration and loading rate

In order to determine the effect of sulfide concentration and loading rate on sulfide removal and denitrification by *Pseudomonas* sp. C27, a set of experiments was conducted in a continuous stirred-tank reactor with a working volume of 500 mL. Liquid medium containing sulfide, nitrate and acetate at the designed concentrations was pumped into the sterilized reactor continuously using a peristaltic pump. Effluent was transferred into an effluent container through an overflow tube. The mixotrophic medium was prepared in a 2-L glass flask and autoclaved for 30 min at 121 ^o^C. Once cooled to room temperature, the medium was purged with filter sterilized nitrogen for 30 min followed by sealing the glass flask with butyl rubber stoppers immediately. Filter sterilized nitrogen was introduced to the flask continuously along the whole system to maintain pressure. Sulfide stock solution (1 M) was then added to achieve the designed concentration and pH was adjusted to 7.5 ± 0.1.

The reactor was charged with prepared mixotrophic medium containing 6.25 mM sulfide, 7.14 mM nitrate and 10.42 mM acetate, and inoculated with 100 mL of a 2-day-old *Pseudomonas* sp. C27 culture. To maintain the anaerobic conditions, filter sterilized nitrogen was introduced into the bioreactor headspace at a low flow rate. Once complete removal of sulfide was achieved, mixotrophic medium containing either 9.38 and 7.14 mM or 14.06 and 25 mM or 17.66 and 27.43 mM sulfide and nitrate (10.42 mM acetate in all medium), respectively, was pumped into the reactor at a flow rate of 5.0 mL h^−1^ and HRT = 36 h. At each bioreactor sufficient time (3–5 residence times) was given for the establishment of steady state conditions^5^. The experiments were carried out at 30 ^o^C. Influent and effluent sampling ports were respectively located at outlet of glass flask (medium container) and between bioreactor and effluent container. Concentrations of sulfide, sulfate, thiosulfate, nitrate, nitrite, and acetate were determined daily. In all cases, analysis of the samples was repeated three times.

### Analytical procedures

The concentrations of sulfate, thiosulfate, nitrate, nitrite and acetate in liquid samples after 0.45 μm filtration were measured using ion chromatography (ICS-3000, Dionex, Bannockburn, IL, USA). Aqueous sulfide was determined spectrophotometrically with N, N dimethyl-p-phenylene diamine^29^. Biomass concentration was monitored indirectly by measuring the protein concentration^22^. Measurement of protein in cell extract was performed by the Lowry method using the bovine serum albumin as the standards.

## Additional Information

**How to cite this article**: Xu, X.-J. *et al.* Characterization of a newly isolated strain *Pseudomonas* sp. C27 for sulfide oxidation: Reaction kinetics and stoichiometry. *Sci. Rep.* **6**, 21032; doi: 10.1038/srep21032 (2016).

## Figures and Tables

**Table 1:** The values of the kinetic parameters for sulfide biooxidation reported in the literature and this study.

Reference	Microbial culture	Maximum specificgrowth rate (μ_m_, h^−1^)	Half saturationconstant (K_S_, mM)	Decay coefficient(k_d_, h^−1^)	Yield coefficient(Y_X/S_, g biomass/mmol sulfide)
This study	*Pseudomonas* sp. C27	0.11	0.68	0.11	0.014
Gadekar *et al.*^19^	*Thiomicrospira* sp. CVO	0.36	1.99	0.0014	0.018^b^
McComas and Sublette^4^	Enrichment dominated by *Thiomicrospira* sp. CVO	0.021	–	–	0.007
Alcantara *et al.*^25^	*Thiobacillus* sp. strain A1	0.1–0.2	0.28	–	0.005–0.010
Sublette and Sylvester^28^	*T. denitrificans*	0.065–0.091	–	–	0.012
Marco de Graaff *et al.*^30^	Enrichment dominated by *Thioalkalivibrio* sp. strain K90-mix	1.48^a^	0.23	–	–

^a^The maximum specific sulfide consumption rate (mmol sulfide mgN^−1 ^h^−1^) is defined by 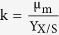.

^b^Yield coefficient calculated assuming that 1 mg ATP corresponds to 1 g dry-weight cell^31^.
